# The Efficacy of Upper-Extremity Elastic Resistance Training on Shoulder Strength and Performance: A Systematic Review

**DOI:** 10.3390/sports10020024

**Published:** 2022-02-14

**Authors:** Rachel C. Seguin, Alan C. Cudlip, Michael W. R. Holmes

**Affiliations:** Department of Kinesiology, Brock University, St. Catharines, ON L2S 3A1, Canada; rs17cq@brocku.ca (R.C.S.); acudlip@brocku.ca (A.C.C.)

**Keywords:** upper extremity, isometric strength, isokinetic strength, muscle, elastic resistance

## Abstract

Elastic resistance exercise is a popular mode of strength training that has demonstrated positive effects on whole-body strength and performance. The purpose of this work was to identify the efficacy of elastic resistance training on improving upper limb strength and performance measures for the shoulder. Seven online databases were searched with a focus on longitudinal studies assessing shoulder elastic training strength interventions. In total, 1367 studies were initially screened for relevancy; 24 full-text articles were included for review. Exercise interventions ranged from 4–12 weeks, assessing pre-/post-strength and performance measures inclusive of isometric and isokinetic strength, 1RM strength, force-velocity tests, and throwing-velocity tests. Significant increases in various isometric strength measures (IR:11–13%, ER:11–42%, FL: 14–36%, EXT: 4–17%, ABD: 8–16%), 1RM strength (~24% in bench press), force-velocities, throwing- and serve-velocities (12%) were all observed. Elastic resistance training elicited positive effects for both strength and performance parameters regardless of intervention duration. Similar significant increases were observed in isometric strength and 1RM strength across durations. Isokinetic strength increases were variable and dependent on the joint velocity conditions. Quantifying the dosage of appropriate exercise prescription for optimal strength and performance gains is inconclusive with this study due to the heterogeneity of the intervention protocols.

## 1. Introduction

Strength training provides a multitude of health and performance benefits. This training involves a diverse range of movements that require the muscles to counteract some form of resistance or force. The use of this training method enables improvements in static and dynamic muscle function, bone strength and formation, joint range of motion, joint stability [1], and athletic performance while decreasing injury risk [2,3,4]. These same benefits hold true across the lifespan, with demonstrated improvements in both young and old participant populations [1,3,4,5,6]. While many strength training modalities are available, differences exist in the volume of research across modalities and the efficacy of their long-term use as a training methodology.

Elastic resistance training (ERT) is a popular method of resistance training. This type of training enables users to perform functional movements in any direction, alternative to traditional free weights, which provide an external force for the muscles against gravity. Upper extremity ERT typically involves attaching the band to a wall or post, standing at a distance to create tension in the band while performing rectilinear, curvilinear, or circular motions with each arm. This can facilitate increases in maximum torque production and stabilization at the shoulder [5,7]. ERT has sport-specific utility; overhead athletes have used elastic resistance to mimic throwing motions including external rotation and abduction movements during warmup [8]. Continued research on elastic resistance has elaborated on muscle activation differences, particularly in posture examinations and the use of single- and dual-vector elastic setups [9,10].

ERT is a viable mode of resistance training to employ in strengthening and rehabilitation programs due to its low cost, versatility, and applicability to all populations. This method of resistance training has received support for its simplicity and feasibility among elderly populations [11,12], effective home-based programs [13,14], and for its usefulness in advanced lifters and athletic populations by providing a varied form of resistance [15,16]. Elastic bands have diverse training applications, including speed and agility training, stretching, plyometric training, and reactive neuromuscular training [17]. The simplicity, versatility, and inexpensiveness of elastic bands for training could combat the commonly perceived barriers to strength training such as the fear of injury, high costs of training equipment, and the intimidation of using fitness facilities [18].

The effects of this type of training on whole-body muscle strength have previously been explored [19,20], but the efficacy of this specific type of training on isokinetic and isometric strength measures of the shoulder remains unknown. Strength gains observed with single-joint and multi-joint elastic resistance exercises have shown to be comparable to that of conventional resistance training [19,21,22,23,24]. The effects of resistance training have been documented to be affected by the sex, health status, and initial strength capability of the user, and should be considered in the context of this treatment method. A comprehensive analysis of elastic training and its effects on shoulder strength would provide clinicians, rehabilitation specialists, and strength and conditioning coaches with information to determine its utility for their clients. The purpose of this review was to assess the current literature on elastic resistance training and collate its findings to determine its efficacy on shoulder strength and performance parameters.

## 2. Materials and Methods

**Figure 1:**
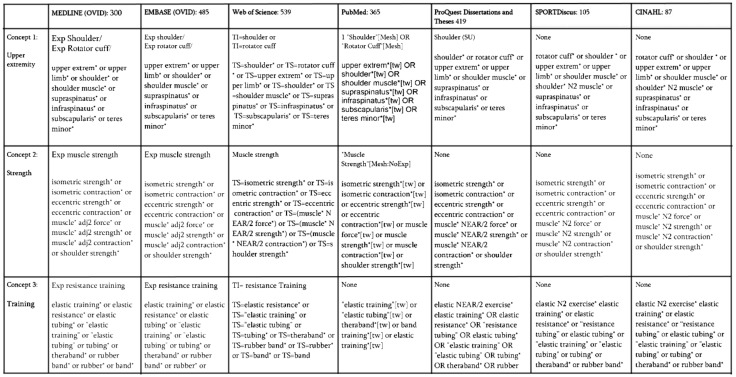
Search string entries for all databases. * represents a wildcard operator to increase the possible number of search terms that contain the preceding letters (e.g. ‘shoulder’ and ‘shoulders’ are both returned by shoulder *). N2 means that the words appear within two words of each other.

A search was devised by considering the main topics of interest and carefully selecting keywords to efficiently extract articles from each database. Seven databases in total were searched on 11 December 2020, including MEDLINE, Embase, Web of Science, PubMed, ProQuest Dissertations and Theses, SPORTDiscus and CINAHL. The search strategy was critiqued and revised by the institutional Library staff to formulate a finalized search string (Figure 1). The search was comprised of a combination of three classifications with their affiliated keywords; these sections were focused on the upper extremity, strength, and performance measures, and the elastic resistance training modality. The study was registered to PROSPERO (ID: CRD42021236849).

All articles were screened for eligibility to be included within this review. Included were randomized control trials, systematic reviews/meta-analyses, cohort studies, and theses. No restrictions on publication dates were applied. A minimum training regimen of twice weekly for 4 weeks with pre- and post- regimen strength or performance metrics was required for randomized control trial inclusion. The strength and performance parameters could include isokinetic or isometric strength assessments, one-repetition maximum (1RM) testing, force-velocity tests, or throwing-velocity tests. The participant population was limited to healthy subjects of all ages. Exclusion criteria involved incorrect study design, patient populations with shoulder pathologies or known adverse health conditions, or outcome measures that did not assess pre- and post-muscular strength.

**Figure 2:**
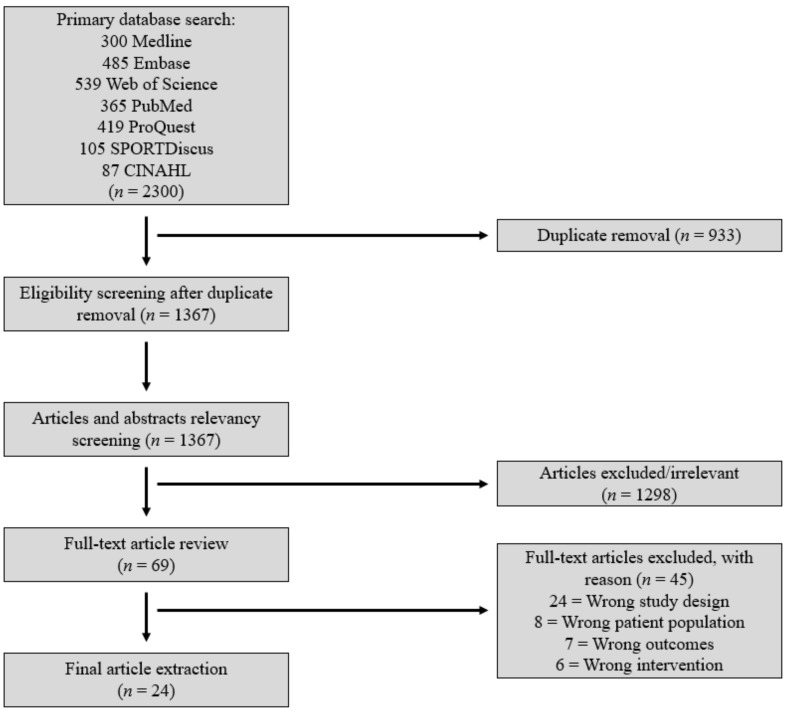
All titles collected through database searches were screened for eligibility; article dispersion is described through this process flowchart.

A multi-step screening process was applied to arrive at the final selection of studies for full-text analysis and data extraction. Initial database searches were conducted by the primary author and were subsequently extracted and screened according to the inclusion/exclusion criteria. The articles were then uploaded to Covidence (Veritas Health Innovation Ltd., Melbourne, Australia) to manage screening processes. Abstract and title screening was conducted first by two independent reviewers, with a third independent reviewer to resolve reviewer decision conflicts. Following the initial screening, eligible articles were assessed using a full-text screen with identical criteria and conflict resolution methods as the first round (Figure 2). All articles that passed the second round of screening were the final studies included for data extraction.

A modified Downs and Black quality checklist [25] was used to assess the methodological quality of each study that was extracted; fourteen criteria from this checklist were utilized. Each study was given a score for each criterion with “yes” = 1, “no/unable to determine” = 0, and a total score out of 14 was yielded. The threshold for adequate quality was a score of 7; any article with a score lower than this was excluded from the review. The ratings of each article are provided in Table 1.

All studies were assessed for potential risks of bias by using the Risk Of Bias In Non-randomised Studies [47] (Table 2). The level of bias assigned to each study was formulated from seven domains: The randomization process, intervention deviations, absence of outcome data, measurements of outcomes, and the reported selection of results. Escalating ratings were described as low (L), moderate (M), serious (S), and critical (C). Bias ratings were totaled based on the highest rating within the seven categories.

The principal summary measure extracted from all included articles was the differences in pre- and post-test mean strength and performance measures. These measures were divided into five main categories, with strength-based measures including isokinetic strength, isometric strength, and 1RM strength, as well as two performance measures of throwing-velocity and force-velocity tests. A percent-change was calculated from the pre-/post-strength and performance measures to normalize increases or decreases of the parameters being measured that occurred over the strength training protocol. This allowed for quantification in strength and performance gains and uniformity of the different variables of performance and strength measurements.

## 3. Results

### 3.1. Study Selection

Collectively, 1367 studies were extracted through initial database retrieval; 24 articles were extracted for assessment following the screening. A detailed flowchart of article screening is detailed in Figure 2. Each article included in the review had data extraction through six key components deemed essential for appropriate analysis of this strength intervention, which included the participant pool, study duration, elastic training intervention exercises, session details, mode of strength measurement, and strength quantification pre- and post-intervention. A full table and the characteristics for each respective study can be found in Appendix A.

### 3.2. Participant Pool

The ages of the participants within the studies were variable, ranging between adolescents >18years of age [26,28,31,32,39,40,46] to elderly patients <65 years of age [33,42]. Some studies assessed strictly males or females [26,27,28,31,32,36,39,40,43,45,46,48,49], one study assessed both and performed a between-subject factor [41], while the rest of the studies assessed both sexes in the same category [7,29,30,33,34,37,38,41,42,44]. The study conducted by Kim et al. [35] did not specify its participants.

### 3.3. Bias and Quality Assessments

The methodological quality of each study was represented by a score out of 14, and an overall bias rating was given for each article. The methodological quality scores and the rating of bias for each article are located in Table 1 and Table 2, respectively. Qualitative scores for all studies were no less than 7, with a range of 7 to 12 (50–86%) and a mean score of 10 [13]. Risk of bias assessments of each article conducted using the ROBINS-I identified one study with serious risk of bias due to deviation from the intended interventions [27]; five studies had a moderate risk of bias in multiple domains, including concerns due to confounding [44], participant selection [7], intervention classification [44,45,46], deviations from intended interventions [41], and missing data [7,45].

### 3.4. Exercise Protocol

The duration of the elastic-training interventions ranged from 4 to 12 weeks. Five studies were 4 weeks in duration [7,30,31,36,38,44], nine studies were 6 weeks in duration [27,34,39,40,42,43,48], four studies were 8 weeks in duration [26,41,44,45], and four studies were comprised of 10–12-week interventions [28,33,37,46,49]. The majority of intervention protocols were three days per week (78%), as one study consisted of two exercise sessions each week [26], and one study with a protocol of five days per week [30]. The most commonly employed elastic resistance bands in the studies assessed in this review were Theraband^®^ at varying resistance levels. Many studies made use of multiple colour-resistance levels as a source of progressive overload over the course of the intervention. Overload was also introduced by increasing band stretch to facilitate increased tension. Three studies did not specify the type of banded resistance used [37,38,39] and one study used an “MVP band”, a circular band that attaches around the wrist rather than being held in the hand [31].

The exercises completed and the angles of resistance of the band were variable. The bands used in nearly all exercise protocols were fixed to a wall or doorknob at hip height or elbow height. Many studies did not specify band tension at the onset of exercise; those that did, started either in a slack or high-tension setting. Specific exercises were generally described as classifications of movements, including abduction exercises, shoulder-retraction exercises, flexion and extension exercises, and internal and external rotation exercises. Appendix A provides specific exercise-session details including repetitions, sets, and the number of sessions per week.

### 3.5. Strength Performance Assessment Results

Various strength and performance measures were assessed within the included studies. Handheld dynamometers and isokinetic dynamometers were the most popular method of measuring upper-extremity strength in the studies included (70%), which measure muscle strength through isometric contraction or specific joint-velocity conditions [50,51]. The most common isokinetic dynamometers used were the Kin-Com^®^ or CYBEX^®^ and were typically employed at joint velocities from 60–240° per second [7,28,39,48,49]. Performance and strength measures collected using 1RM tests included variations of lying bench press, dumbbell pullover, seated row, shoulder press, and shoulder abduction [26,33,43,45]. Force-velocity tests were performed on a Monark cycle ergometer [26] and throwing- and serving-velocity evaluations were conducted with the use of a radar gun [26,31].

Time-dependent measures are illustrated in Appendix A. Increases in various strength and performance measures were observed regardless of the length of the exercise intervention. Significant increases of 7–42% improvement were observed in multiple isometric strength measures across all studies. Studies measuring internal and external rotation isometric strength observed significant increases in both parameters, with increases in internal and external rotation isometric strength increasing by 11.2–13.5 and 11.0–42.3% across studies, respectively [29,30,38,44]. Increases in isometric flexion and extension were observed across all studies, with significant (*p* < 0.05) increases of 14.7–36.0% and 4.7–17.1%, respectively. [30,34,35,37,52]. The few studies that assessed isometric abduction observed both significant (*p* < 0.05) [30,37] and insignificant increases [41,52] ranging from 8–16%. All studies that evaluated 1RM strength found increases that were significant, regardless of the duration of the strength training program [26,43,45]. An average increase of 24% was observed for lying and seated variations of the bench press. Richards and Dawson [43] found significant increases of 11.4–25.2% in 1RM shoulder flexion and abduction strength after six weeks of training, as other studies observed increases in various concentric lifts [26,33,45]. Throwing and serving velocity increases were observed over variable intervention durations. Escamilla et al. [31] found significant increases in baseball-throwing velocities (3.9%) after a four-week protocol, as significant increases in both force-velocities and throwing-velocities (W_peak_ increases of 36%) were observed by Aloui et al. [26].

The changes that occurred for isokinetic strength were much more variable over time. Significant increases were observed by Baker [27] and Batalha [28] in external rotation at 180 degrees/s (4.2–4.4%), while Page et al. [48] observed decreases of 14.8% during isokinetic diagonal movement patterns at 180°/s.

Decreases in strength were observed at lower internal rotation joint velocities (60°/s) by Baker and Batalha, with decreases of 2.1–2.6%. Similar decreases (2.7–4.6%) were found in eccentric internal and external rotation strengths after eight weeks by Sugimoto et al. [44]. Opposingly, Treiber [7] and Knerr [36] observed increases in all joint velocity conditions for both external and internal rotation (2.3–21.2%).

Collectively, four studies reported significant increases in isometric internal rotation (IR) strength [30,34,38,44], three of which also reported similar significant increases in external rotation (ER) isometric strength [29,30,38]. Four studies also found significant (*p* < 0.05) increases of 8.1–42.3% in isometric flexion, extension, and abduction [30,34,35,37]. Increases in isokinetic strength were observed variably across all studies. Two studies observed increases in isokinetic internal rotation strength at higher joint velocities (+12.13% at 180 degrees/s [36], +21.24% at 300 degrees/second [7]), although Batalha et al. [28] did not observe this same effect and only observed significant increases of ER isokinetic strength at 180 degrees/s.

## 4. Discussion

The primary aim of this review was to examine the effects of longitudinal ERT programs on shoulder strength and performance measures. Although the effects of elastic resistance training on whole-body strength gains have previously been explored, the specific effects of this mode of training on the upper extremity remained unclear. A consensus from the included studies identified statistically significant increases in external rotation, internal rotation, flexion and extension, and abduction strength of the shoulder following varied ERT programs. Secondly, this mode of strength training yielded significant increases in 1RM strength—particularly in the lying and seated bench press—and performance measures including throw and serve velocities that increased by ~11% over a six- to eight-week regimen [26,31,32]. Lastly, there are some positive effects of ERT on isokinetic strength, though, these results are less conclusive due to varying observations at different joint velocities. Some studies observed increases at all joint velocities [27,36] and increases in both eccentric and concentric ER and IR strengths [36,40,44] while some studies observed decreases in certain variables of isokinetic strength, such as Page [48] and Mascarin [40] who observed decreases at higher joint velocities at 180°/s and 240°/s, respectively. Collectively, the increases observed in performance measures and strength variables, particularly in peak torque, 1RM strength, force velocities, and throwing velocities identify ERT as a viable mode of resistance training for eliciting observable strength and performance gains in individuals participating in longitudinal strength training programs. These 1RM increases were observed across a diverse participant pool; the five studies that had 1RM as an outcome metric included university-aged participants of both sexes [33], national-level handball players [26], and post-menopausal women [45]; these groups collectively saw reported 1RM increases of 13–24%. These 1RM tests were completed using non-elastic equipment such as CYBEX machines, but strong relationships between submaximal elastic resistance and estimated maximal strength have been quantified and could be used in future research designs [53]. Due to the lack of homogeneity in the exercise interventions of the studies included and the range of initial strength and normalized strength increases, an optimal prescription of upper extremity training with elastic resistance cannot be concluded, and further research is needed.

Previous studies have concluded that elastic devices utilized in training can produce strength gains that are equivalent to those observed with free weights and conventional-device training [19,54]. ERT has also previously been proven to be effective in improving whole-body strength and function in the elderly [3,55]. This is similar to the findings within this review, as a minimum of a four-week elastic resistance training program has demonstrated improvements in isometric strength and 1RM strength, in addition to other performance measures and isokinetic strength variables. The ease of access and versatility of this type of training paired with the positive effects observed among studies included in this review indicated this training may be useful for clinicians and trainers to implement as a longitudinal program to aid in the strength and performance of their clients.

There are limitations to be considered for the intervention methods employed. The strength training protocols for studies included in this review were not uniform. The intervention protocols varied considerably in length, where strength and performance gains could be attributed to different factors. The increases observed for shorter intervention durations were likely due to neurological adaptations such as increased motor unit recruitment and neural drive to the working muscles, whereas longer intervention durations likely elicited strength gains from both central and peripheral adaptations [56,57]. As muscle volume was not measured in the examined studies, the noted strength gains could have resulted from a combination of factors. There is a paucity of documented strength changes with elastic resistance training in populations beyond healthy, university-aged participants, despite the simplicity and feasibility of its use [1,3,11]. The papers included in this review focused on strength. Thus, studies with a rehabilitation focus that may not have documented a strength outcome were not included. This work highlights the need to continue to examine the effects of this training method on expanded populations, including those with differences in initial strength or health, as well as understanding differences across sex and age groups. Variability existed in the exercises completed and their accompanying sets and repetitions, making inferences regarding appropriate dosing of an ERT regimen to elicit optimal strength and performance gains difficult. The starting tension of the elastic band was inconsistent across studies, as some programs instructed participants to begin with minimal resistance or tension, while others had participants initiate movements at a distance from the wall that removed slack in the band. Discrepancies between the slack length or length of stretch could be a cause for patient variability and a lack of codified information on resistance levels within these studies obscure recommendations. The initial resting length of the bands is a crucial component to consider, as the resistance that is generated throughout any movement completed with the band is dependent upon the relative stretch of the material. Many of the intervention protocols implemented in each study did not provide details on the monitoring of exercises being completed, the set-up of the bands, or the progression of the coloured resistance levels over time. While progression over the training period occurred in most included studies, it was unclear what percentage of the maximum the elastic training represented. Lastly, variability existed in the methodology of measuring strength variables, particularly in isometric and isokinetic strength [58]. These strength measures were assessed at different internal and external rotation angles, and different joint velocities ranging from 60–240°/s, which may have confounded results.

## 5. Conclusions

Longitudinal elastic resistance training protocols involving upper extremity movements such as external rotation, internal rotation, abduction, and elevation elicit increases in strength and performance for the general healthy population. The considerable heterogeneity of the exercise regimens and methods of assessing strength make it difficult to firmly conclude the types of exercises and protocols that should be employed in training and clinical settings to elicit the most observable strength and performance enhancements. The documented changes in strength may represent a portion of the progressive changes seen in these groups, and additional reviews focusing on the potential existence of technique, range of motion, or fatigue resistance changes when using this exercise methodology would be beneficial. This enforces the need for more rigorous studies that follow a more standardized exercise regimen and protocol of measuring strength and performance parameters.

## Figures and Tables

**Table 1:** Modified Downs and Black methodological quality assessment ^1^.

Article	1	2	3	4	6	7	9	10	11	12	16	18	20	26	Total
Aloui et al. (2019) [26]	1	1	0	1	1	1	1	1	0	0	1	1	1	1	11
Baker, J.A (1992) [27]	1	1	0	1	1	1	0	0	U	U	1	1	1	U	8
Batalha et al. (2018) [28]	1	1	1	1	1	1	1	1	U	U	1	1	1	U	11
Bussey, H.I (2000) [29]	1	1	1	1	1	1	1	0	0	0	1	1	1	U	10
Behm, D.G. (1991) [6]	1	1	1	1	1	1	0	0	U	0	1	1	1	1	10
Cho et al. (2018) [30]	1	1	1	1	1	1	0	0	0	0	1	1	1	U	9
Escamilla et al. (2010) [31]	1	1	1	1	1	1	1	1	U	U	1	1	1	1	12
Fernandez et al. (2013) [32]	1	1	1	1	1	1	1	1	0	0	1	1	1	1	12
Gibson, T.S. (2002) [33]	1	1	1	1	1	0	1	1	0	0	1	1	1	U	10
Hibberd et al. (2010) [34]	1	1	1	1	1	1	0	1	0	0	1	1	1	U	10
Kim et al. (2018) [35]	1	1	0	1	1	1	0	0	U	U	1	1	U	1	8
Knerr, S.A (1995) [36]	1	1	0	1	1	1	1	0	0	0	1	U	1	1	9
Lima et al. (2018) [37]	1	1	1	1	1	1	0	0	U	U	1	1	1	1	10
Magnus et al. (2014) [38]	1	1	0	1	1	1	1	1	U	U	1	1	1	U	10
Markovic et al. (2016) [39]	1	1	0	1	1	1	1	0	U	U	1	1	1	1	10
Mascarin et al. (2017) [40]	1	1	1	1	1	1	1	1	0	0	1	1	1	U	11
Page et al. (1993) [17]	1	1	0	1	1	1	1	1	0	0	1	1	1	1	11
Picha et al. (2019) [41]	1	1	1	1	1	1	1	1	U	U	1	1	1	U	11
Pourtaghi et al. (2017) [42]	1	1	1	1	1	1	1	1	1	1	1	1	1	U	13
Richards, J.A. (2009) [43]	1	1	1	1	1	1	1	0	0	0	1	U	1	1	10
Sugimoto et al. (2006) [44]	1	1	1	1	1	1	1	0	U	U	1	1	1	1	11
Thiebaud et al. (2013) [45]	1	1	0	1	1	1	1	1	U	U	1	1	1	U	10
Treiber et al. (1998) [7]	1	1	0	1	1	1	0	0	0	0	1	1	1	1	9
Vaezi et al. (2015) [46]	1	1	0	1	1	0	1	0	U	U	1	1	0	U	7

^1^ Scoring: 1 = yes, 0 = no, ‘U’ = unclear (equates to zero).

**Table 2:** Risk of bias assessments following criteria from ROBINS-I tool ^2^.

Article	1	2	3	4	5	6	7	Total
Aloui et al. (2019) [26]	L	L	L	L	L	L	L	L
Baker, J.A (1992) [27]	L	L	L	S	L	L	M	S
Batalha et al. (2018) [28]	L	L	L	L	L	L	L	L
Bussey, H.I (2000) [29]	L	L	L	L	L	L	L	L
Behm, D.G. (1991) [6]	L	L	L	L	L	L	L	L
Cho et al. (2018) [30]	L	L	L	L	L	L	L	L
Escamilla et al. (2010) [31]	L	L	L	L	L	L	L	L
Fernandez et al. (2013) [32]	L	L	L	L	L	L	L	L
Gibson, T.S. (2002) [33]	L	L	L	L	L	L	L	L
Hibberd et al. (2010) [34]	L	L	L	L	L	L	L	L
Kim et al. (2018) [35]	L	L	L	L	L	L	L	L
Knerr, S.A (1995) [36]	L	L	L	L	L	L	L	L
Lima et al. (2018) [37]	L	L	L	L	L	L	L	L
Magnus et al. (2014) [38]	L	L	L	L	L	L	L	L
Markovic et al. (2016) [39]	L	L	L	L	L	L	L	L
Mascarin et al. (2017) [40]	L	L	L	L	L	L	L	L
Page et al. (1993) [17]	L	L	L	L	L	L	L	L
Picha et al. (2019) [41]	L	L	L	M	L	L	L	M
Pourtaghi et al. (2017) [42]	L	L	L	L	L	L	L	L
Richards, J.A. (2009) [43]	L	L	L	L	L	L	L	L
Sugimoto et al. (2006) [44]	M	L	M	L	L	L	L	M
Thiebaud et al. (2013) [45]	L	L	M	L	M	L	L	M
Treiber et al. (1998) [7]	L	M	L	L	M	L	L	M
Vaezi et al. (2015) [46]	L	L	M	L	L	L	L	M

^2^ Bias Domains: (1) Bias due to confounding; (2) bias in selection of participants to the study; (3) bias in classification of interventions; (4) bias due to deviations from intended interventions; (5) bias due to missing data; (6) bias in measurement of outcomes; (7) bias in the selection of the reported result. Total score is the highest risk value across domains. L = low; M = moderate; S = serious risk of bias.

## Data Availability

Not applicable.

## Appendix A

**Table A1:** Summary of studies extracted, including performance measures, the sample population, type and length of training, exercise-session details, measurements of strength, and the quantification in pre-/post-strength and performance.

Outcome Measure	Study	Participants	Type of Elastic Resistance Training	Length of Study	Movement (Exercises Completed)	Session Details	Strength Measurement	Quantified Strength and Performance After Protocol	Summary
Isokinetic Strength	Treiber et al. [7]	22 subjects females/males Mean age: 21.2 yrs/old	Theraband^®^ attached to wall at roughly elbow height. Progressed from red, green, blue.	4 weeks	Internal and external rotation exercises Two sets of 20 repetitions at slow continuous speed, two sets at a quick functional speed	Regular practice sessions 5×/week. Participated in sessions 3×/week for 4 weeks	Concentric maximal torque of ER and IR, Cybex 6000 Isokinetic Dynamometer-Tested at 120°/s and 300°/s	EG peak torque: IR 120°/s: 6.67%↑ IR 300°/s: 21.24%↑ ER120°/s: 5%↑ ER 300°/s: 15.56%↑	EG exhibited significantly greater increases in peak torque to body weight for both IR and ER torque at 300°/s (*p* < 0.01)
	Markovic et al. [39]	40 experienced junior male athletes. 17.2 ± 1.0 years	No specification of ER used. Resting length 1.5 m, coefficient of elasticity 133 N/m. One end fixed behind subject at hand level	6 weeks	Subjects performed six sets of ten repetitions of jab punch each with the instruction to reach a target	Intervention added to the regular training routine. ERT applied 3×/week for 6 weeks	Kin-Com isokinetic dynamometer—rapidly exerted maximum force	Vpeak shoulder: (F = 17, *p* < 0.01; ES = 0.59) Main effect of time (F = 167, *p* < 0.01; ES = 0.82)	Vpeak of the shoulder revealed a significant time-group interaction. Significant main effect of time (pre-post) (*p* < 0.01)
	Batalha et al. [28]	25 young male swimmers -Land group (*n* = 13) Mean age: 13.52 ± 0.92 yrs	Thera-Band^®^ red elastic band to initially placed around the wrists. Progression occurred when subject could do 30 repetitions by upgrading colours.	10 weeks	(1) Upper-limb abduction and ER (2) Abduction to 160 degrees (3) 90° flexion/abduction ER	3×/week, progression every two weeks 3 sets of each exercise	IR and ER isokinetic strength by isokinetic dynamometer Exerting maximal effort (peak torque was evaluated during the performance of three repetitions at 60º/s and 20 repetitions at 180º/s)	Dominant arm PT change (Nm) ER 60°/s: 1.15 IR 60°/s: −2.10 ER 180°/s: 4.15 IR 180°/s: −0.18 Non-dominant: ER 60°/s: 0.99 IR 60°/s: −2.59 ER 180°/s: 2.53 IR 180°/s: 0.009	Significant increases in ER of the dominant shoulder at 180°/s (*p* < 0.05) and in unilateral ratios
	Knerr, S.A. [36]	14 males from Ball State University between the ages of 18 and 24.	Theraband^®^ anchored at waist 8m from wall. Progressed by stepping further from wall & increasing colour-resistance.	4 weeks	Exercised the internal and external rotators muscles of their experimental shoulder	3 sets of 8 repetitions using the maximum amount of resistance that could be repeated 10 times. 3×/week.	Cybex 6000 isokinetic dynamometer Testing conducted at 60° and 180°/s	PT % change: ER 60°/s = 2.9% ER 180°/s = 4.7% IR 60°/s = 2.3% IR 180°/s = 12.3%	Increase in percent improvement in IR Increases in peak torque production at 180 deg/s
	Baker, J.A. [27]	22 female subjects Mean age of 25.9 years	Theraband^®^ attached to door knob. 8 colours of theraband used to progress	6 weeks	Concentric and eccentric contractions of the ER’s of the shoulder, elbow at 90 degrees	Three sets of 10 repetitions per day. 3×/week for 6 weeks. 3 s per contraction	Isokinetic testing at 60°/s and 180°/s angular velocities using Cybex II isokinetic machine.	60°/s: 5.1%↑ 180°/s: 4.41%↑	No significant difference for 60°/s Significant difference in strength at 180°/s t = 3.04
	Page et al. [17]	Twelve collegiate baseball pitchers All males	Theraband attached to the wall even with the iliac crest, 3 ft from the origin with no slack in the band. Began with light (yellow band), to red, green, and blue.	6 weeks	Exercises: Circumduction Abduction Biceps Curls Triceps Extensions Standing supraspinatus “Emptycan” Posterior Cuff ER Horizontal Abduction	Subjects performed three sets of 10 repetitions/day. Each session added five more repetitions, up to 25 repetitions. Exercises were performed 3×/week	KIN-COM^®^ isokinetic dynamometer Subjects instructed to perform repetitions at 50%, 75% and 100% “perceived maximum”	Theraband group 60°/s: % diff =19.8 ↑ Theraband group 180°/s: % diff =14.8↓	No difference at 180°/s; (decreased) Theraband was effective at 60°/s in functional eccentric strengthening of posterior rotator cuff in the pitching shoulder.
	Mascarin, N.C. et al. [40]	Total: 25 female handball players age: 15.3 ± 0.9 yrs Experimental group for dominant arm (*n* = 8) Non-dominant (*n* = 5)	Four colour levels (blue, black, silver, and gold) of Theraband used at a wall distance of 0.70 m, with the band stretched to minimal resistance	6 weeks/18 sessions	Two exercises for ER muscle (1) standing position with 90 degrees of shoulder abduction and elbow flexion and (2) neutral shoulder, elbow flexed at 90 10 repetitions ×3 sets with the blue band (light resistance)	3×/week STP with Thera-Band^®^ exercise program was implemented for the experimental Group Progression via increases in RPE, repetitions, distance from wall to 2.00 m	- Isokinetic dynamometer - Tested with five repetitions for concentric action at 60° and 240°/s and eccentric action at 240°/s.	Dominant arm: Concen IR 60°/s: 4%↑ Concen ER 60°/s: 16.79%↑ Concen ER 240°/s: 6.28%↓ Eccen ER 240°/s: 1.96%↓ Non-dominant arm: Concen IR 60°/s: 1.02%↓ Concen ER 60°/s: 15.86%↓ Concen ER 240°/s: 5.56%↓ Eccen ER 240°/s: 26.4%↑	Significant increase in ER peak torque and total work values in concentric contraction at 60°/s No changes in eccentric ER peak torque at 240°/s
	Sugimoto, D. et al. [44]	40 subjects: Elastic band (*n* = 12), mean age 24.3 yrs M/F = (3/9)	Four colours of Theraband used (thin/yellow, medium/red, heavy/green, and extra heavy/blue resistance). Intensity increased by standing further away from fixed wall at elbow level	8 weeks	resisted shoulder internal and external rotation exercises with repetitions	3 sets × 20 reps Progression occurred via distance from wall and elastic used 3×/week	Isometric, concentric, and eccentric muscle strength of the internal and external shoulder rotators was measured by a KinCom isokinetic dynamometer	IR: Concen. 60°/s: 7.08% ↑ Concen. 120°/s: 3.36%↑ Eccen. 60°/s: 6.91%↑ Eccen.120°/s: 4.58%↓ ER: Concen. 60°/s: 2.81%↑ Concen. 120°/s: 6.0%↑ Eccen. 60°/s: 2.56% ↑ Eccen. 120°/s: 2.68%↓	Significant group X test interaction for peak external rotation concentric torque at 120°/s
	Behm, D. [6]	31 male subjects, mean age 20.4 years	Surgical tubing tied into loops against a straight-backed chair	10 weeks	Shoulder press	3 sets of 10 repetitions, 1 s per repetition	A Cybex II isokinetic dynamometer for shoulder abduction torque was assessed at 60/120/180/240/300 deg/s; 1RM shoulder press on Universal machine	14.7% increase in isokinetic shoulder strength; 13.8% increased in Universal shoulder press strength	Increase in shoulder strength; no indication of movement-specific or velocity-specific training responses
Isometric Strength	Bussey, H.I. [29]	34 subjects, 27 male, 7 female ages: 18 to 45 years	Thera-Band^®^ tied into loop, tied to a fixed doorknob. Progressed through yellow, red, green, blue, black, to grey bands	6 weeks	Rockwood Five protocol shoulder strengthening exercises (flexion, extension, ER, IR, abduction movements)	Five exercises performed three times a day, 5 repetitions of each, held for count of 5. 3×/week	A MicroFET 2^®^ hand held dynamometer was used to assess strength measurements of shoulder external rotation	Mean ER 0°: 32.74% ↑ Mean ER 45°: 35.01% ↑ Mean ER 70°: 42.34% ↑	Statistically significant within-group interactions. Significant increases in ER strength after 6 weeks
	Pourtaghi, F. et al. [42]	70 elderly Mean age: 69.7 ± 6.1 yrs-Males (*n* = 22) Females (*n* = 48)	Three colours of Theraband^®^, red (medium), green (heavy), and blue (extra heavy) used	6 weeks	Lower- and upper-extremity resistance training with Thera-Band	Two thirty-minute sessions a week for six weeks	Standard push-pull dynamometer	Right arm: diff: 20.65%↑ Left arm: Diff: 19.47%↑	Mean scores of muscular strengths were significantly higher pre-post
	Sugimoto, D. et al. [44]	40 subjects: Elastic band (*n* = 12), mean age 24.3 yrs M/F = (3/9)	Four colours of Theraband used (thin/yellow, medium/red, heavy/green, and extra heavy/blue resistance). Intensity increased by standing further away from fixed wall	8 weeks	Resisted shoulder internal and external rotation exercises with repetitions	3 sets × 20 reps Progression occurred via distance from wall and elastic used 3×/week	IRand ER isometric and isokinetic strength tests at 60°/s and 120°/s. Measured by a KinCom isokinetic dynamometer	Maximal isometric IR: 65° of ER: ↑11.03 10° of IR: ↑12.94 Maximal isometric ER: 65° of ER: ↑28.92 10° of IR: ↑8.69	A significant group X test interaction for maximal isometric IR torque at 10° of IR and maximal isometric ER torque at 65° of ER
	Magnus, C.R.A., et al. [38]	23 participants aged 50.0 + 9.0 years, both males (*n* = 11), females (*n* = 12)	Four different strengths of resistance tubing (no specification of type). Yellow (4–5 lbs resistance), red (9–10 lbs), blue (12 lbs), and black (16 lbs).	4 weeks	Tubing for maximal shoulder ER, IR, scaption, retraction, and flexion Dynamic and isometric exercises	3×/week for 4 weeks. 10–15 repetitions to failure for each set and leave a minute rest between sets	Handheld dynamometry Maximal isometric shoulder strength (ER, IR, scaption) Elbow bent at 90° in seated position	ER: Diff: 11.02%↑ IR: Diff: 12.10%↑	Significant time main effects for external and internal rotation of the trained subjects
	Lima, F.F. et al. [37]	29 total ETG (*n* = 10) Over 45 years old, males and females	Five models of elastic tubing used. All tubes were connected to a specific chair with length and position adjusted for each trained muscle group.	12 weeks	Movements performed in the following order: shoulder abduction, elbow flexion, shoulders flexion, knee extension and knee flexion	12 weeks (3×/week) with recuperative intervals of 48 to 72 h between sessions	Handheld digital dynamometer (Force Gauge^®^, model FG-100 kg, USA) in the dominant UL: shoulder flexors & abductors and elbow flexors	∆%0–12 Weeks: Shoulder abduction = +16% Shoulder flexion= +36%	Significant increases in both shoulder abduction and flexion after 12 weeks in the ETG group
	Picha, K.J. et al. [41]	73 total Elastic band group (*n* = 36) 23 females, 10 males aged: 32 ± 15yrs	Thera-Band CLX Consecutive Loops used. 3 different colours of resistance	8 weeks	Three exercises performed - Shoulder abduction - Shoulder external rotation - Shoulder extension	Exercises completed 3×/week for 8 weeks	Maximal isometric strength measures were obtained bilaterally using a dynamometer completing shoulder abduction and shoulder ER	Strength changes over 8 weeks Males shoulder abduction: right = 5.2%↑ *p* = 0.480 left = 6.2%↑ *p* = 0.505 Males shoulder ER: right = 2.8%↑ *p* = 0.739 left = 2.5%↑ *p* = 0.816 Females shoulder abduction: right = 2.7%↑ *p* = 0.826 left = 2.5%↑ *p* = 0.357 Females ER: right = 1.9%↑ *p* = 0.851 left = 2.2%↑ *p* = 0.510	Shoulder strength increased at a rate of approximately 0.5% body mass (BM) per week
	Hibberd, E.E. et al. [34]	37 Division I collegiate swimmers Intervention group (*n* = 20) 10 F, 10 M Mean age: 19.2 ± 1.2	Theraband used, colour-resistance progression used but not specified.	6 weeks	scapular retraction (Ts), with upward rotation (Ys), downward rotation (Ws), shoulder flexion, low rows, throwing acceleration and deceleration, scapular punches, shoulder IR & ER at 90° abduction	3×/week for 6 weeks	Isometric strength measured via handheld dynamometer	(% Body Mass/% change) Flexion: 2.0 ± 5.0 Extension: 4.7 ± 6.9 ER:1.6 ± 3.8 IR:4.0 ± 7.1	Subjects in the intervention group gained 2.0% of their body mass in shoulder-flexion strength and 1.7% in shoulder-abduction strength shoulder-extension and internal-rotation strength significantly increased
	Cho et al. [30]	28 subjects post-dropout EBG (M/F): 8/6 Mean age: 29.0 (3.6) yrs EBG-DOG (M/F): 9/5 Mean age 29.6 (3.3) yrs	Extra heavy (blue color) of Thera-Band^®^ used. The length of the Thera-Band^®^ was held at a constant 1.52 m.	4 weeks	EBG performed shoulder flexion, extension, abduction, adduction, horizontal abduction/adduction, and internal/external rotation EB-DOG performed exercises for 15 min and the double oscillation exercise in three planes of motion (frontal, sagittal, and transverse), using a Bodyblade	EBG: 30 min/session, 5×/week, for four weeks. EB-DOG:15 min/session, 5×/week, for four weeks.	Shoulders examined for flexion, extension, abduction, adduction, horizontal abduction/adduction, internal/external rotation, and protraction, using a handheld dynamometer.	Strength change % EBG: Flexion: 18.73↑ Extension: 17.05↑ ER: 14.48↑ IR: 13.48↑ Strength change % EB DOG: Flexion: 14.02↑ Extension: 16.88↑ ER: 8.09↑ IR: 9.15↑	Significant increase in all categories of shoulder muscle strength for both groups
	Kim, M. et al. [35]	19 subjects Stabilization group (*n* = 9) Mean age: 20.7 (1.6) yrs	Blue coloured Theraband used	4 weeks	15 min of shoulder strengthening exercises and 15 min of shoulder stretching exercises on pectoralis minor muscle TheraBand colored blue was used	The main exercise was repeated 10 times in a set of 10 s and the rest time was 2-min between the sets The groups performed each exercise for 40 min, 3×/week, for 4 weeks.	A functional rehab system measured isometric strength, shoulder flexion, extension, abduction, horizontal abduction and adduction	Flexion: 14.69%↑ Extension: 8.96%↑ Abduction: 11.05%↑ Adduction: 7.96%↑	Significant increase in the left and right directions of the LOS Significant increase in the maximal flexion strength
1RM	Vaezi et al. [46]	33 male teenaged volleyball players average age 16.4 ± 1.21 years Elastic group (*n* = 9)	Theraband^®^ was used	12 weeks	Bench press, shoulder, front thigh, leg curl & leg press machine	2 sessions per week for 12 weeks, 10–12 repetitions of each exercise	1 RM (bench press, shoulder abduction) Anaerobic Wingate, Sargent jump test	No concrete results reported. Only graphs.	
	Richards, J.A. [43]	Fourteen female athletes (*n* = 14) Aged 18 to 30 years.	Theraband^®^ was attached under foot at a given length to ensure 200% resistance (measured according to individual’s 10RM that was calculated prior)	6 weeks	Conventional program: shoulder flexion and isolated shoulder abduction with tubing. Multidirectional exercise regimen: ‘‘8′’ and ‘‘N’’ movement arcs	3 sets of 10 repetitions, 3×/week for 6 weeks	1RM protocol—shoulder flexion and abduction	Graphs with no exact numbers	Significant 1RM improvements for both experimental groups for dominant and non-dominant arm flexion and abduction
	Thiebaud et al. [45]	Postmenopausal women (61 ± 5 yrs) 14 participants completed the study	Theraband^®^ force elongation for elastic bands was at an estimated ~10%–30% of each participant’s 1RM.	8 weeks	upper body exercises (seated chest press, seated row, seated shoulder press) followed by lower body exercises (knee extension, knee flexion, hip flexion, hip extension).	Training sessions 3×/week for 8 weeks, 48 h between sessions	1RM testing → The first testing session included supine leg press, supine chest press, right and left hip extension, and right and left hip flexion second testing session included shoulder press, right and left knee extension, seated row and right and left knee flexion	Strength for chest press: Pre: 254 (54) kg Post: 291 (61) kg %diff: ↑13.58 Seated row: Pre: 376 (42) kg Post: 398 (43) kg %diff: ↑11.37 Shoulder press: Pre: 264 (50) kg Post: 278 (59) kg %diff: ↑5.17	Strength significantly increased for chest press, seated row, and shoulder press
	Aloui et al. [26]	30 male healthy handball players Single national-level Tunisian team	Theraband^®^ at 200–250% elongation. Three different levels of resistance used: black (Special Heavy), silver (Super Heavy) and gold (Maximum Heavy).	8 weeks	Four exercises: shoulder internal rotation at 90° abduction, elbow extension, shoulder horizontal adduction, and shoulder sagittal adduction). 1:30 s rest interval given between sets. All exercises performed with maximal effort.	2×/week for 8 weeks, 30- minute sessions. Experimental group replaced a part of their standard regimen with the elastic band training program	Force-velocity test via Monark cycle ergometer Throwing velocity recorded by digital video camera 1RM testing via bench press and pullover	1RM strength: bench press pre = 66.4N post = 83.1N %diff: ↑22.34 Pull over Pre = 25.4N Post = 36.5N %diff: ↑35.86	Large significant increases in 1RMPO (d = 1.90) and 1RMBP (d = 1.51) for experimental group (EG)
	Gibson, T.S. [33]	41 total subjects Elastic training group (*n* = 20) mean age of 73.47 (±6.23) Males (*n* = 6) Female (*n* = 15)	Theraband used. Began with yellow band (lightest), progressed intensity if to complete more than 15 repetitions in the third set of each exercise	12 weeks	Seven exercises (3 lower body, 4 upper body): Seated chest press Seated row Shoulder press Hammer curl	The home-based training group (*n* = 20) exercised using elastic bands and body weight for resistance, 3×/week for 12 weeks completing three sets of eight to 12 repetitions for each of the seven exercises.	1RM testing Two measurements taken for both pre and post pectorals, deltoids, rhomboids, trapezius, biceps, triceps	Gained strength percentages: Seated row = 24.12 Shoulder press = 17.35 Seated bench press = 25.15	Significantly improved on all of the five strength measures: seated row, shoulder press, seated bench press, and hammer curl.
Throwing/ Serving Velocity	Aloui et al. [26]	30 male healthy handball players Mean age: 18.3 ± 0.8 years A single national-level Tunisian team	Theraband^®^ at 200–250% elongation. Three different levels of resistance used: black (Special Heavy), silver (Super Heavy) and gold (Maximum Heavy).	8 weeks	Four exercises: shoulder internal rotation at 90° abduction, elbow extension, shoulder horizontal adduction, and shoulder sagittal adduction). 1:30 s rest interval given between sets. All exercises performed with maximal effort.	2×/week for 8 weeks, 30-min sessions. Experimental group replaced a part of their standard regimen with the elastic band training program	Force-velocity test via Monark cycle ergometer Throwing velocity recorded by digital video camera 1RM testing via bench press and pullover	Standing throwing velocity: ↑22.6%	The increase in peak power was accompanied by large and significant increases in all three types of throwing velocity
	Escamilla, R.F. et al. [31]	Thirty-four youth baseball players (11–15 years of age) Training group (*n* = 17), males	‘‘MVP Band,’’ system used that attaches to the wrists	4 weeks	17 upper extremity resistance exercises Exercises included: chest flies, internal and external rotation exercises, diagonal flexion patterns, etc.	75 min in duration 3×/week for 4 weeks. Two experienced trainers in the training group 20–25 repetitions per exercise	Jugs Tribar Sport radar gun (Jugs Pitching Machine Company, Tualatin, OR, USA) Five maximum effort-throwing trials	pre-test = 25.1m/s post-test = 26.1 m/s % diff = ↑3.90 *p* = 0.004	Throwing velocity increased significantly in the training group
	Fernandez et al. [32]	Thirty competitive healthy nationally ranked malejunior tennis players (mean age 14.2 ± 0.5 yrs)	Theraband used (red and green band), attached to wall	6 weeks	Nine upper extremity strength exercises: elbow extension, rowing, ER variations, shoulder abduction, diagonal pattern flexion, reverse throw, forward throw, wrist flexion extension	2 sets of 20 repetitions, with 45 s rest between exercises 3 sessions (60–70 min) weekly	Stalker Professional Sports Radar used to measure serve velocity	Serve velocity (km/h) Pre: 150.3 Post: 157.9 % diff: ↑4.93	Significant improvement in the serve velocity for the training group (*p* = 0.0001)
Force-velocity	Vaezi et al. [46]	33 male teenaged volleyball players average age 16.4 ± 1.21 years Elastic group (*n* = 9)	Theraband^®^ was used.	12 weeks	Bench press, shoulder, front thigh, leg curl & leg press machine	2 sessions per week for 12 weeks, 10–12 repetitions of each exercise	1 RM (bench press, shoulder abduction) Anaerobic Wingate Sargent jump test	No concrete results reported. Only graphs.	
	Aloui et al. [26]	30 male healthy handball players <18 years old (a national-level Tunisian team)	Theraband^®^ at 200–250% elongation. Three different levels of resistance used: black, silver, and gold.	8 weeks	Four exercises with maximal effort: shoulder internal rotation at 90° abduction, elbow extension, shoulder horizontal adduction, and shoulder sagittal adduction.	2×/week for 8 weeks, 30-min sessions.	Force-velocity test via Monark cycle ergometer Throwing velocity recorded by digital video camera 1RM testing via bench press and pullover	Wpeak (kg) pre = 5.20 post = 7.51 ↑36.35%	Statistically significant increases for power were observed (d = 1.77) for EG
